# Comparative Analysis of Conventional and Digital Microscopy for Counting Mitotic Figures in Cutaneous Neoplasms of Dogs and Cats

**DOI:** 10.3390/ani16081268

**Published:** 2026-04-21

**Authors:** Larissa G. A. Moreira, Lucas R. Souza, Nayara F. Paula, Taismara S. Oliveira, Ayisa R. Oliveira, Taryn A. Donovan, Christof A. Bertram, Tatiane A. Paixão, Renato L. Santos

**Affiliations:** 1 Departamento de Clínica e Cirurgia Vetetrinárias, Escola de Veterinária, Universidade Federal de Minas Gerais, Belo Horizonte 31270-901, Minas Gerais, Brazil; gianninilari@gmail.com (L.G.A.M.); lucasdosreisdesouza1@gmail.com (L.R.S.); nay.ferreiradepaula@gmail.com (N.F.P.); ayisa.rodrigues@gmail.com (A.R.O.); 2 Hermes Pardini do Grupo Fleury, Vespasiano 33200-000, Minas Gerais, Brazil; tsimasoli@gmail.com; 3 Schwarzman Animal Medical Center, New York, NY 10065, USA; donovanta@gmail.com; 4 Department of Biological Sciences and Pathobiology, University of Veterinary Medicine Vienna, 1210 Vienna, Austria; christof.bertram@vetmeduni.ac.at; 5 Departamento de Patologia, Instituto de Ciências Biológicas, Universidade Federal de Minas Gerais, Belo Horizonte 31270-901, Minas Gerais, Brazil; tatipaixaoufmg@gmail.com

**Keywords:** mitotic counts, mitosis, neoplasm, dog, cat

## Abstract

The use of digitized slides for histopathological diagnosis has become common in veterinary pathology, and the validation of diagnostic techniques that are extrapolated from the evaluation of glass slides is needed. The goal of this study is to evaluate the efficiency of counting mitotic figures in physical glass slides and digitized slides of cutaneous tumors of dogs and cats. The mitotic count was performed by three pathologists on glass and digitized slides of ninety skin tumors, including 30 squamous cell carcinomas in dogs and cats, 30 mast cell tumors and 30 soft tissue tumors in dogs. An additional assessment of cellular proliferation was performed with immunohistochemistry for Ki67. Spearman’s correlation for the mean count of mitotic figures between the three observers on physical and digitized slides demonstrated a strong positive correlation for squamous cell carcinomas and mesenchymal tumors and a moderate correlation for mast cell tumors. Inter-observer agreement was moderate between the two methods. In conclusion, the results found suggest that digitized slides can be used reliably for mitotic figure counting in cutaneous neoplasms in small animals, without compromising their classification or prediction of prognosis.

## Simple Summary

Digital pathology allows veterinary pathologists to examine microscope slides as high-resolution images on a computer instead of using traditional glass slides. Before this approach can be widely adopted, it is important to confirm that diagnoses made using digital images are as reliable as those made with a microscope. This study compared the counting of mitotic figures (dividing cells), an important indicator of tumor growth and aggressiveness, in glass slides and digitized slides from skin tumors of dogs and cats. Three pathologists evaluated 90 tumors, including squamous cell carcinomas, mast cell tumors, and soft tissue tumors. The researchers also measured cell proliferation using a laboratory test called Ki67 immunohistochemistry. The results showed that mitotic counts obtained from digital slides were very similar to those obtained from glass slides, especially for squamous cell carcinomas and soft tissue tumors. Although some differences were observed between observers, overall agreement between the two methods was good. These findings indicate that digitized slides can be reliably used to evaluate skin tumors in small animals, supporting the use of digital pathology without affecting tumor classification or prognosis prediction.

## 1. Introduction

The use of digitized slides has markedly increased in veterinary pathology over the past few years, particularly in high-throughput, large diagnostic laboratories and academic institutions. Digital pathology is a very convenient tool since it facilitates archiving, may allow a more accurate diagnosis with the use of software, and expedites second-opinion consultations or remote discussion of histopathology cases [1]. Despite these advances, diagnostic and classification criteria traditionally adopted while examining glass slides (GSs) still need to be validated when using digitized slides (DSs) [2,3].

Over the past two decades, numerous validation studies have been carried out in human medicine, generating relevant data on various aspects of histopathology with the goal of assessing the concordance between GSs and DSs, thus validating diagnoses based on DSs [4,5,6,7,8,9]. However, similar validation studies are still scarce in veterinary medicine. There are a couple of cytopathological studies, comparing morphological diagnoses between GSs and DSs [10,11], while another study addressed the comparison of regions of interest (ROIs) between GSs and DSs [12]. Previous studies assessing the histopathological diagnosis of canine cutaneous neoplasms comparing GSs and DSs are scarce [13,14].

Mitotic count is considered a well-established approach for estimating cell proliferation in neoplastic tissues, commonly considered for malignancy classifications of neoplasms and, in some cases, represents an important prognostic parameter [15,16,17,18]. In human medicine, good results have been reported regarding mitotic counting (MC) in DSs, indicating that MC performed on GSs or DSs yield similar results [4,6], but similar studies are scarce in veterinary medicine [14]. Therefore, the goal of this study was to compare the efficiency of conventional and digital microscopy for counting mitotic figures in cutaneous neoplasms of dogs and cats.

## 2. Materials and Methods

A total of 90 randomly selected (based on the order they were submitted) cases of surgical biopsies of cutaneous tumors of dogs and cats received at the Universidade Federal de Minas Gerais (Brazil) between 2021 and 2022 were included in this study. The cases were previously diagnosed by a veterinary pathologist and selected to compose three groups: 30 epithelial tumors (squamous cell carcinomas—SCCs) including 19 from dogs and 11 from cats; 30 mesenchymal tumors (soft tissue tumors—STTs) including 24 from dogs and 6 from cats; and 30 canine mast cell tumors (MCTs), high or low grade, only from dogs (samples were randomly included regardless of their grade). From each case, a representative glass slide (GS) and its corresponding digital slide (DS) were selected for analysis.

**Figure 1:**
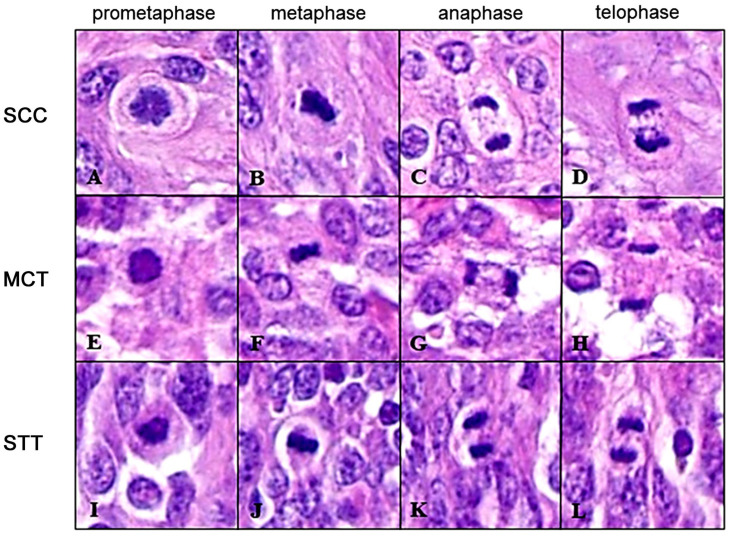
Representative mitotic figures at various stages visualized in digitized slides used in this study showing, respectively, prometaphase (**A**), metaphase (**B**), anaphase (**C**) and telophase (**D**) in squamous cell carcinoma (SCC); prometaphase (**E**), metaphase (**F**), anaphase (**G**) and telophase (**H**) in mast cell tumors (MCTs); and prometaphase (**I**), metaphase (**J**), anaphase (**K**) and telophase (**L**) in soft tissue tumors (STTs). Hematoxylin and eosin, 40×.

Paraffin-embedded tissues were sectioned using a microtome (3.0 µm thick sections) and mounted onto glass slides for histology and onto glass slides with double grip (silanized and polarized) (EasyPath, Indaiatuba, Brazil) for immunohistochemistry. Histologic slides were automatically stained with hematoxylin and eosin (H&E) and coded (from 1 to 90); then they were scanned at a single focus point at 40× magnification resulting in a resolution of 0.26 µm/pixel (Aperio GT 450, Leica Biosystems, Nussloch, Germany). MC was performed by three observers (OB1, OB2 and OB3) with a similar degree of training (recently completed or last-year residency training on veterinary pathology). For MC, observers were instructed to look for hotspots (areas of highest mitotic activity), avoiding necrotic, cystic, markedly inflamed areas, and histological artifacts. MC occurred at two time points over a total period of nine weeks, when each observer received 12 or 24 slides per week, including GSs and DSs, with a minimal interval (wash-out period) of a two weeks, as recommended by the American College of Pathologists [19]. Identification of mitotic figures was based on their morphology (Figure 1), carefully avoiding impostor mitotic figures as described by Donovan et al. (2021) [15]. The area used for counting was 2.37 mm^2^, consistent with 10 standard high-power fields [16], calculated using the numerical factor of the eyepiece lens of physical microscopes in higher magnification fields (40× objective, FN 22). The digital calculation was done as proposed by Meuten et al. (2016) [16], using monitor measurements to define the number of fields and the Aperio ImageScope software (version 12.4) to read the digital slides.

Immunohistochemistry: For the immunohistochemistry, slides were deparaffinized and rehydrated in a series of decreasing ethanol concentrations (100–70%). Antigenic retrieval was performed using pressured heat (approximately 120 °C for 10 min) in a pH 6.0 citrate buffer solution (DakoCytomation, Glostrup, Denmark). Endogenous peroxidase blocking was performed using an Endogenous Peroxidase Blocking Solution (DakoCytomation), and nonspecific protein blocking was performed using a 6.0% skimmed milk solution. The primary antibody (Monoclonal Mouse Anti-Human Ki67 Antigen, DakoCytomation) was incubated at a concentration of 1:50 overnight (12–16 h) in a dark humid chamber at 4 °C. The Linker (Flex+ Mouse LINKER, DakoCytomation) was used for 30 min and then the HRP-conjugated secondary antibody (DakoCytomation) for 30 min. The immunoreaction was revealed using the chromogen 3,3–diaminobenzidine tetrahydrochloride (Liquid DAB + Substrate Chromogen System, DakoCytomation) for 10 to 20 min in cases of SCCs and for 5 to 10 min in cases of MCTs and mesenchymal tumors. The slides were counterstained with hematoxylin, dehydrated in increasing ethanol concentrations (70–100%) for 3 min each, followed by xylene for 3 min, and mounted with coverslips. The basal layer of the epidermis and the hair follicles were used as internal positive controls. The slides were scanned in the same way as the H&E-stained slides, and the proliferation index (PI) was obtained by consensus of the three observers, using the digitized version of each one of the cases and counting the positive-stained nuclei after counting a total of 500 nuclei. The same cells were concomitantly evaluated by the three observers and considered as having positive staining or not; any intensity of nuclear staining was considered as positive. The three observers met at a single computer, and fields of interest were evaluated at the highest possible magnification until the cell count reached 500; areas of necrosis with inflammatory infiltrate or with any confounding bias were excluded.

Statistical analyses: For the statistical analyses, the Friedman test was performed followed by the Dunn test to compare the medians between GSs and DSs by observer and by type of tumor, and differences were considered significant when *p* < 0.05. For correlation analysis, Spearman’s correlation test was performed contrasting MC in GSs and DSs considering various parameters: all tumors, tumor types, inter- or intra-observer variability. To remove the individual variability of the observers, the arithmetic means between the readings of the three of them for each case and slide modality were calculated. Then, Spearman’s correlation was calculated between the means of total glass and digital counts and for each tumor type. Additionally, Spearman’s correlation test was also performed with the PI found in digitized Ki67 immunohistochemistry slides and the MC per observer and tumor types. For the interpretation of all the correlation tests, the following parameters were used: r ≤ 0.3 or r ≤ −0.3 interpreted as a very weak correlation; 0.3 ≥ r ≤ 0.5 or −0.3 ≥ r ≤ −0.5 as a weak correlation; 0.5 ≥ r ≤ 0.7 or −0.5 ≥ r ≤ −0.7 as a moderate correlation and r ≥ 0.7 or r ≥ −0.7 as a strong correlation. These statistical analyses were made using GraphPad Prism 8 software (GraphPad Prisma software, Inc 8.0.1, San Diego, CA, USA).

Finally, SCC counts were divided using the separatrix method of tertiles, the MCT counts according to the 2-tier grading system (≤6 or ≥7) [20] and the STT counts according to a previously described grading system [21,22], wherein Score 1 = 0–9 mitotic figures, Score 2 = 10–19 mitotic figures and Score 3 > 19 mitotic figures, all per 10 high-power fields (2.37 mm^2^). A weighted Kappa test was used to assess agreement between GSs and DSs according to Landis and Koch (1977) [23], in which the strength of concordance was categorized as mild (0.00–0.20); regular (0.21–0.40); moderate (0.41–0.60); substantial (0.61–0.80); and almost perfect agreement (0.81–1.00). In addition, an intraclass correlation coefficient (ICC) analysis was performed (absolute agreement type; two-way random model) [24], and a Bland–Altman plot was generated.

## 3. Results

**Figure 2:**
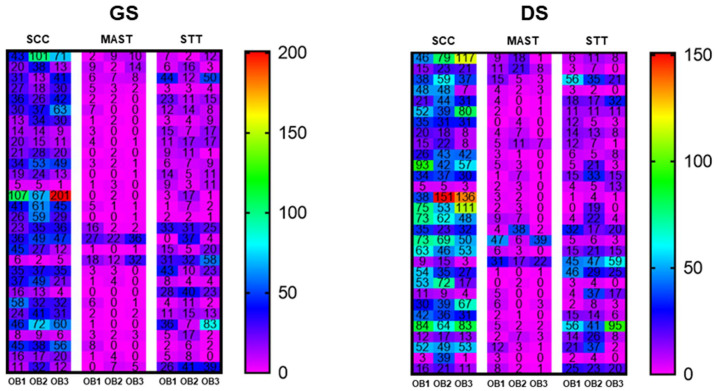
Heatmap with mitotic counts in cases of squamous cell carcinoma (SCC), mast cell tumors (MCTs) and soft tissue tumors (STTs) performed by three different observers (OB1, OB2 and OB3) on glass (GS) or digitized (DS) hematoxylin and eosin-stained slides. Data are presented in individual values per case, per evaluator and per tumor. Sidebar represents mitotic count absolute values.

**Figure 3:**
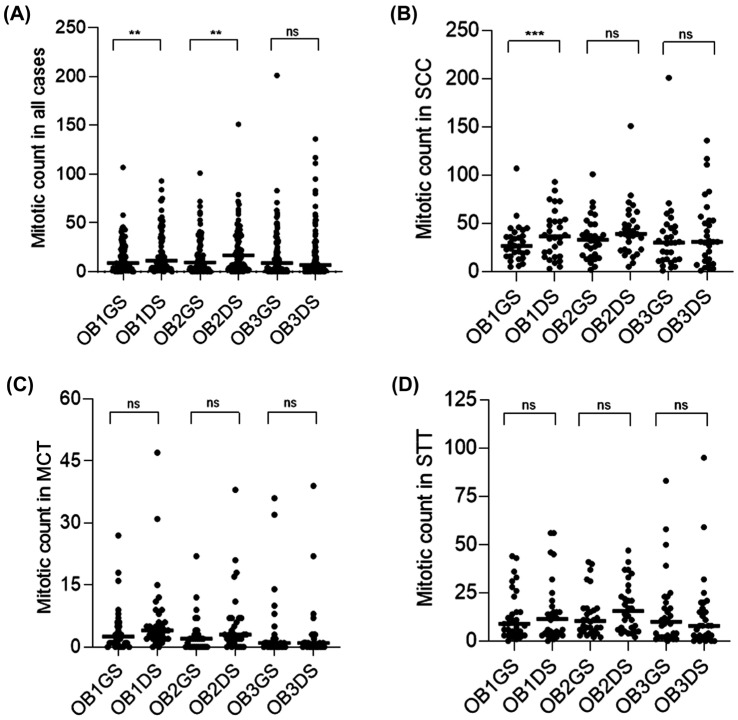
Mitotic count performed by three different observers (OB1, OB2 and OB3) on glass (GS) and digitized (DS) hematoxylin and eosin slides. (**A**) Comparison of intra-observer mitotic count on GSs and DSs in all 90 cases. OB1 and OB2 show significant statistical difference between GSs and DSs. (**B**) Comparison of intra-observer mitotic count on GSs and DSs in SCC. OB1 shows significant statistical difference between GSs and DSs. (**C**,**D**) Comparison of intra-observer mitotic count on GSs and DSs in MCTs and STTs. There is no statistically insignificant difference for any observer. Dots represent individual values and the bars the medians values. Statistical differences are represented by ** (*p* < 0.01), *** (*p* < 0.001) (ns = statistically insignificant difference). Friedman’s test followed by Dunn’s test.

MC results by tumor type (squamous cell carcinomas (SCCs), mast cell tumors (MCTs) and soft tissue tumors (STTs)), observer (OB1, OB2, and OB3) and type of slide (GS or DS) are presented in Figure 2 as a heatmap with their individual and absolute values. A total of 4617 mitotic figures were counted on GSs and 5545 mitotic figures on DSs, considering all cases and all observers. The total GS and DS analysis showed no statistical difference between medians. Considering all 90 cases (30 SCCs, 30 MCTs and 30 STTs), MC was significantly different between GSs and DSs for OB1 and OB2, who had higher MC medians for DSs than for GSs (*p* < 0.01), whereas no significant difference was observed for OB3 (Figure 3A). When compared with the tumor type, only SCCs had a significant difference between GSs and DSs only for OB1 (*p* < 0.001); the other tumor types and observers did not have significant differences in MC comparing GSs and DSs (Figure 3).

**Figure 4:**
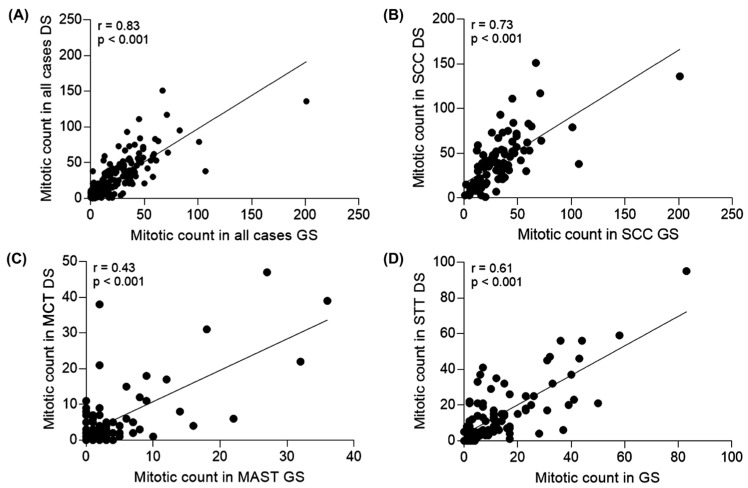
(**A**) Spearman correlation analysis between arithmetic mean of mitotic counts in all 90 cases: 30 squamous cell carcinoma, 30 mast cell tumors and 30 soft tissue tumors, evaluated by three different observers on glass (GS) and digitized (DS) hematoxylin and eosin slides. The value of r = 0.83 demonstrates a strong positive and significant correlation (*p* < 0.001) between the two forms of assessment. (**B**) Spearman correlation analysis in squamous cell carcinomas (SCCs), mast cell tumors (MCTs) (**C**) and soft tissue tumors (STTs) (**D**) evaluated by three different observers on glass (GS) and digitized (DS) hematoxylin and eosin slides. The r value demonstrates a strong positive correlation (r = 0.73) for cases of SCC, a weak positive correlation (r = 0.43) for MCTs and a moderate positive correlation (r = 0.61) for cases of STT tumors, and all were significant (*p* < 0.001).

Correlation analysis between GSs and DSs, including all 90 tumors and all three observers resulted in r = 0.83, indicating a strong, positive, and significant correlation (*p* < 0.001) (Figure 4A). When analyzing each tumor type separately, the correlation between GSs and DSs was strong and positive (r = 0.73; *p* < 0.001) for SCC cases (Figure 4B); weak and positive (r = 0.43; *p* < 0.001) for MCTs (Figure 4C); and moderate and positive (r = 0.61; *p* < 0.001) for STTs (Figure 4D).

The inter-observer correlation analysis (Spearman) demonstrated that, for DSs, there was a strong positive and significant correlation (*p* < 0.001) for MC in SCCs among all observers (Table 1). In contrast, for MCTs, correlations were not significant between OB1 × OB2 and OB2 × OB3, whereas a weak, positive and significant correlation was observed in OB1 × OB3 (*p* < 0.05). Regarding STTs, there were moderate, positive, and significant correlations between OB1 × OB2 and OB2 × OB3 (*p* < 0.001) and a strong positive and significant correlation in OB1 × OB3 (*p* < 0.001) (Table 1). A similar analysis of GSs demonstrated a moderate positive and significant correlation between OB1 × OB2 and strong positive and significant correlations of MC between OB2 × OB3 and OB1 × OB3 in SCCs (*p* < 0.001). In cases of MCTs, there was no significant correlation between OB1 × OB2, while there were weak, positive, and significant correlations between OB2 x OB3 and OB1 × OB3 (*p* < 0.05). Regarding STTs, no significant correlations were found between OB1 × OB2 and OB2 × OB3, whereas a strong, positive, and significant correlation was found in OB1 × OB3 (*p* < 0.001) (Table 1).

Intra-observer MC correlation between GSs and DSs (Table 2) was strong, positive, and significant in SCCs for OB1 and OB3, and it resulted in a moderate, positive, and significant correlation for OB2 (*p* < 0.001). In cases of MCTs, there was a weak, positive, and significant correlation between MC in GSs and DSs for OB1 (*p* < 0.05), a moderate, positive, and significant correlation for OB3 (*p* < 0.001), and no significant correlation for OB2. Regarding STTs, a strong, positive, and significant correlation was observed for OB1 and OB3 (*p* < 0.001), and no significant correlation was observed for OB2 (Table 2).

**Figure 5:**
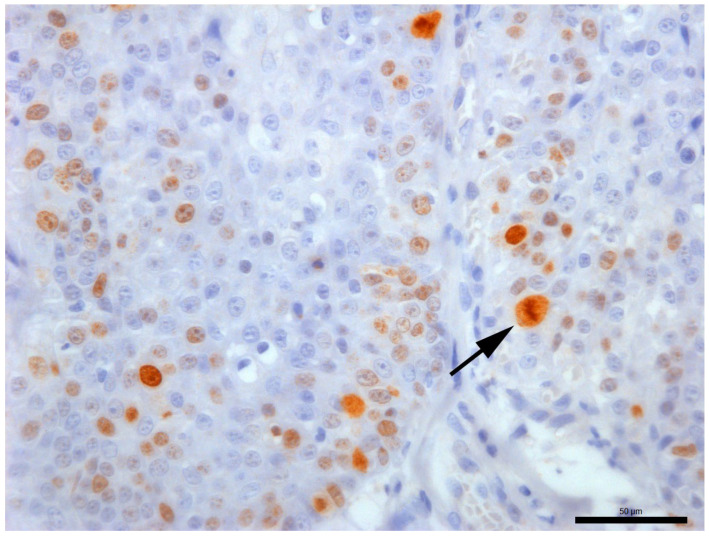
Representative immunohistochemistry for detection of Ki67 in a case of squamous cell carcinoma. Many cells with nuclear immunostaining, including cells morphologically in mitosis (arrow). Anti-Ki67 immunohistochemistry, 40×.

Correlation between the consensus digital proliferation index based on Ki67 immunohistochemistry (Figure 5) and MC per observer and tumor type on GSs had one single significant correlation, which was moderate (0.41) for OB1 in MCTs (*p* < 0.05) (Table 3). In DSs, OB2 obtained a moderate and significant correlation (0.40) for SCC tumors (*p* < 0.05) (Table 3). Using the mean MC of the three observers for GSs and DSs, and the Ki67 digital MI consensus, the correlation was moderate for both (0.65 and 0.66) and significant (*p* < 0.001).

**Figure 6:**
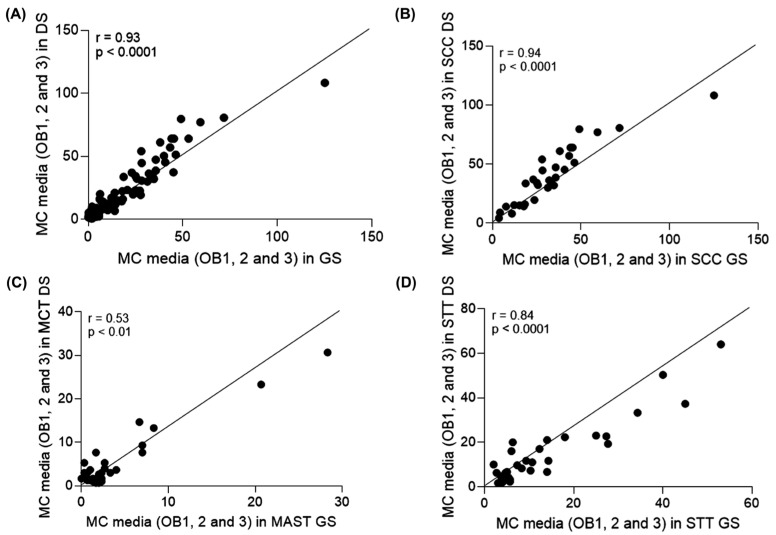
(**A**) Spearman correlation analysis between the mean MC of the three observers in all 90 cases on DSs and GSs, with r = 0.93, a strong positive and significant correlation (*p* < 0.001) between the two forms of assessment without the individuality effect of the observers. (**B**–**D**) Spearman correlation analysis between the mean MC of the three observers in all 90 cases on DSs and GSs, per type of tumor (**B**) SCCs, (**C**) MCTs and (**D**) STTs. The r value demonstrates a strong significant positive correlation (r = 0.94) for cases of SCC and STTs (r = 0.84), *p* < 0.0001. For MCTs, a moderate significant positive correlation was found (r = 0.53, *p* < 0.01).

Considering MC from all observers for all tumor types, there was a strong, positive (r = 0.93) and significant (*p* < 0.0001) correlation between MC in glass and digital slides (Figure 6A). When analyzing each tumor type separately, there was a strong, positive (r = 0.94) and significant (*p* < 0.0001) correlation between MC from observers in GSs and DSs in cases of SCC (Figure 6B). For MCTs, the correlation was moderate, positive (r = 0.53) and significant (*p* < 0.01) (Figure 6C), and for STTs, it was strong, positive (r = 0.084) and significant (*p* < 0.0001) (Figure 6D).

The weighted Kappa analysis indicated the agreement between DS × GS was moderate for SCCs (0.59), MCTs (0.44) and STTs (0.51). Considering the grading of Kiupel et al. (2011) [20] based on the mitotic count of MCTs, they agreed on the same tumor grading in DS × GS in 93.33% (28/30) of the cases, and the two discordant cases were high grade in DSs and low grade in GSs. SCC does not have a grading system that could perform an analysis similar to that of MCTs, and, despite having a grading system, STTs cannot be evaluated like MCTs due to a score that associates the number of mitoses with the necrosis assessment, with the mitosis category having three scores, which increases the variability of analysis when compared with MCTs.

**Figure 7:**
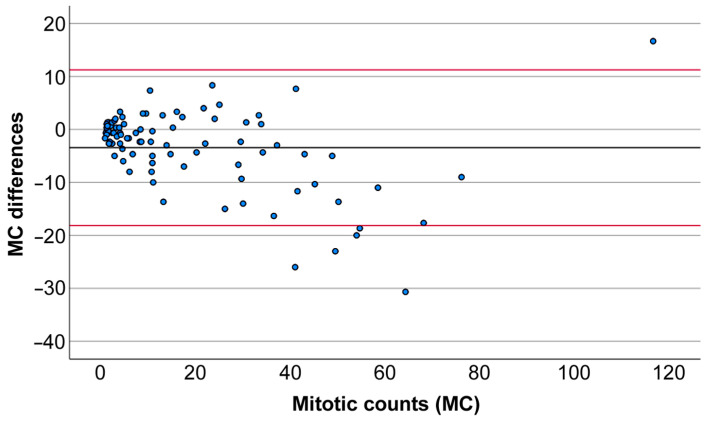
Bland–Altman plot: X-axis is the mean of mitotic count (MC) of the glass slides (GSs; mean value of the 3 observers) and digital slides (DSs; mean value of the 3 observers) of each case (*n* = 90); Y-axis indicates differences between the mean between GSs and the correspondent DSs of the 3 observers (*n* = 90). The black line indicates the average, and the red line indicates coefficient of variation 95%. Less than zero indicates a higher count on the DSs, and greater than zero indicates a higher count on the GSs.

In addition, an intraclass correlation coefficient (ICC) analysis was performed (absolute agreement type; two-way random model) [24], with the aim of evaluating the agreement of the three observers in the mitotic count of the 90 tumors. The results indicated a high inter-observer agreement in GSs (ICC = 0.904; 95% CI = (0.86–0.93), F (89, 178) 10.448, *p* < 0.001) and in DSs (ICC = 0.897; 95% CI = (0.85–0.93), F (89, 178) 9.745, *p* < 0.001). A Bland–Altman plot comparing MC in GSs and DSs by all three observers is presented in Figure 7.

## 4. Discussion

These results indicated good correlations or agreement between the MC in GSs and DSs for cutaneous tumors in dogs and cats. When considering all observers and tumor types together, the correlation between MC obtained from GSs and DSs was strong and significant. However, when tumor types were analyzed separately, correlations varied from weak for MCTs, to moderate for STTs and strong for SCCs. The correlation between GSs and DSs using the mean MC of the observers for each modality per tumor type and individual cases presented an increased, also strong and significant result and was also better correlated between the different types of tumors, ranging from moderate for MCTs to strong for STTs and SCCs. These findings highlight the impact of individual variability on MC. Even though the observers had similar levels of training, differences in familiarity with the modalities under analysis, particularly digital practice and individual backgrounds cannot be excluded [25]. Nevertheless, the inter-observer correlation was higher in the DSs. For both GSs and DSs, correlation was strongest in SCC tumors, whereas MCTs showed the weakest in both modalities, although GSs presented slightly better agreement, with two significant correlations between observers.

MC values in the heatmap demonstrated greater colorimetric heterogeneity in some cases of SCC, in GSs and DSs, in contrast to more homogeneous results observed in MCT and STT cases. This is likely due to SCC histological features since this type of tumor is known to display a more variable number of mitotic figures across the histological section, especially among the less differentiated tumors [26]. However, these considerations did not seem to affect the analyses; the intra-observer correlation was strong for two of the three observers and moderate for the other. Additionally, the overall MC correlation between GSs and DSs for this type of tumor was significant and strong, with moderate Kappa agreement. When considering the mean MC of the three observers, the correlation between GSs and DSs was even stronger and remained significant.

It is well known that intracytoplasmic granules in mast cells can interfere with nucleus visualization and, consequently, the identification of mitotic figures [27]. This may explain the weak correlation observed in the total comparison between the two modalities and the moderate correlation when using the mean MC. Regarding intra-observer correlations, MCTs showed the poorest results, with only two significant analyses, both varying between weak and moderate correlations. These findings may be attributed to the low MC amplitude, as MCTs often present values ranging from zero to two, which may contribute to discrepancies in statistical analyses. Despite the lower individual correlations, the concordance test indicated moderate agreement for MCTs. The choice of the cutoff point for performing the Kappa concordance test was based on the well-consolidated grading system, widely used in veterinary medicine [20]. The low MC limits between high and low grades in MCTs and its prognostic implications played a crucial role in defining these cutoffs. This approach aimed to assess its applicability and clinical impact in veterinary practice, and the results suggest that it is appropriate for these cases.

STTs, previously named soft tissue sarcomas, comprise a group of mesenchymal-origin neoplasms that can originate from various cell types [21], which may present distinct histological features and varying mitotic activity. However, all types of STTs are classified in the same manner by a grading system, initially developed for human pathology, for which the MC is one of the three criteria [21,22,28,29]. The correlation between the two modalities using individual MC was moderate, whereas using the average MC between glass and digital slides resulted in a strong correlation. Similarly, intra-observer results showed that two of the three observers found a strong and significant correlation. Agreement for this type of neoplasm was assessed using the previously mentioned grading system as a cutoff point, revealing moderate agreement, comparable to that observed in MCTs and SCCs.

Absence of strong correlations between the proliferation index based on Ki67 expression and MC was expected, as Ki67 immunohistochemistry detects cells in all mitotic phases [30].

Regarding the validation of MC in digitized slides, there are still no specific guidelines for human or veterinary pathology, and the current diagnostic criteria are based on GS analyses [6]. The evaluation of MC in DSs can be challenging, due to the absence of micrometric control (fine focus) and due to limited image resolution [15]. The MC on DSs in this study was higher than the MC on physical slides, for the same cases, by almost a thousand mitotic figures, which contrasts the findings of previous studies, in which lower MCs were reported in DSs [4,6,13,31].

In general, manual recording of the MC can be a laborious and tiring task [32] and there may be a varying degree of individual fatigue per pathologist at the time of reading [6]; these also should be considered as possible biases for both GSs and DSs. Furthermore, as the purpose of this work was to simulate the MC on a daily basis, only the size of the area in which count should be performed was defined (2.37 mm^2^), so that the hotspots likely varied, mainly between observers [33] but also within observers, considering the two modalities under analysis, which can be considered as responsible for part of the variation found in the inter- and intra-observer correlation tests [14].

## 5. Conclusions

In conclusion, MC in skin neoplasms of dogs and cats had moderate-to-high agreement when comparing evaluations performed on GSs and DSs. Squamous cell carcinomas and sarcomas demonstrated greater reliability and agreement in results. Although some MCTs exhibited discrepant MC, they remained classified within the same prognostic grading system in 93.3% of the cases (28/33).

## Figures and Tables

**Table 1:** Inter-observer Spearman correlation of the MC in digital slides (DSs) and glass slides (GSs) between the three observers (OBs).

**Digital Slides (DSs)**	**OB1 × OB2 (r)**	**OB2 × OB3 (r)**	**OB1 × OB3 (r)**
Squamous Cell Carcinoma	0.72 ***	0.70 ***	0.72 ***
Mast Cell Tumors	0.34	0.36	0.49 *
Soft Tissue Tumors	0.67 ***	0.66 ***	0.78 ***
**Glass Slides (GSs)**	**OB1 × OB2 (r)**	**OB2 × OB3 (r)**	**OB1 × OB3 (r)**
Squamous Cell Carcinoma	0.68 ***	0.75 ***	0.77 ***
Mast Cell Tumors	0.16	0.39 *	0.46 *
Soft Tissue Tumors	0.30	0.25	0.85 ***

* *p* < 0.05 and *** *p* < 0.001.

**Table 2:** Intra-observer Spearman correlation of the MC in digital slides (DSs) and glass slides (GSs) per tumor type.

DS × GS	OB1 (r)	OB2 (r)	OB3 (r)
Squamous Cell Carcinoma	0.72 ***	0.69 ***	0.82 ***
Mast Cell Tumors	0.37 *	0.13	0.62 ***
Soft Tissue Tumors	0.72 ***	0.19	0.76 ***

* *p* < 0.05 and *** *p* < 0.001.

**Table 3:** Spearman correlation between the consensuses of digital proliferation index based on Ki67 immunohistochemistry and MC per observer (OB) and tumor type on GSs and DSs.

**GSs**	**OB1 × Ki67 (r)**	**OB2 × Ki67 (r)**	**OB3 × Ki67 (r)**
Squamous Cell Carcinoma	0.2535	0.3153	0.2522
Mast Cell Tumors	0.4085 *	0.08935	0.1817
Soft Tissue Tumors	0.1738	0.0504	0.0132
**DSs**	**OB1 × Ki67**	**OB2 × Ki67**	**OB3 × Ki67**
Squamous Cell Carcinoma	0.2926	0.4045 *	0.2242
Mast Cell Tumors	0.2605	0.2978	0.3497
Soft Tissue Tumors	0.01573	0.2167	0.3071

* *p* < 0.05.

## Data Availability

Data is contained within the article.

## References

[1] Bertram C.A., Klopfleisch R. (2017). The pathologist 2.0: An update on digital pathology in veterinary medicine. Vet. Pathol..

[2] Pantanowitz L. (2010). Digital images and the future of digital pathology. J. Pathol. Inform..

[3] Bertram C.A., Aubreville M., Donovan T.A., Bartel A., Wilm F., Marzahl C., Assenmacher C.-A., Becker K., Bennett M., Corner S. (2022). Computer-assisted mitotic count using a deep learning-based algorithm improves interobserver reproducibility and accuracy. Vet. Pathol..

[4] Al-Janabi S., Van Slooten H.J., Visser M., Van Der Ploeg T., Van Diest P.J., Jiwa M. (2013). Evaluation of mitotic activity index in breast cancer using whole slide digital images. PLoS ONE.

[5] Koch L.H., Lampros J.N., Delong L.K., Chen S.C., Woosley J.T., Hood A.F. (2009). Randomized comparison of virtual microscopy and traditional glass microscopy in diagnostic accuracy among dermatology and pathology residents. Hum. Pathol..

[6] Lashen A., Ibrahim A., Katayama A., Ball G., Mihai R., Toss M., Rakha E. (2021). Visual assessment of mitotic figures in breast cancer: A comparative study between light microscopy and whole slide images. Histopathology.

[7] Nielsen P.S., Lindebjerg J., Rasmussen J., Starklint H., Waldstrøm M., Nielsen B. (2010). Virtual microscopy: An evaluation of its validity and diagnostic performance in routine histologic diagnosis of skin tumors. Hum. Pathol..

[8] Williams B.J., Hanby A., Millican-Slater R., Nijhawan A., Verghese E., Treanor D. (2018). Digital pathology for the primary diagnosis of breast histopathological specimens: An innovative validation and concordance study on digital pathology validation and training. Histopathology.

[9] Lempp C., Arms S., Bertram C.A., Klopfleisch R., Igl B.W., Hezler L., Nolte T., Pohlmeyer-Esch G. (2024). A minimal approach to demonstrate concordance of digital and conventional microscopy in toxicologic pathology. Toxicol. Pathol..

[10] Bonsembiante F., Martini V., Bonfanti U., Casarin G., Trez D., Gelain M.E. (2018). Cytomorphological description and intra-observer agreement in whole slide imaging for canine lymphoma. Vet. J..

[11] Bonsembiante F., Bonfanti U., Cian F., Cavicchioli L., Zattoni B., Gelain M.E. (2019). Diagnostic validation of a whole-slide imaging scanner in cytological samples: Diagnostic accuracy and comparison with light microscopy. Vet. Pathol..

[12] Blanchet C.J.K., Fish E.J., Miller A.G., Snyder L.A., Labadie J.D., Avery P.R. (2019). Evaluation of region of interest digital cytology compared to light microscopy for veterinary medicine. Vet. Pathol..

[13] Bertram C.A., Gurtner C., Dettwiler M., Kershaw O., Dietert K., Pieper L., Pischon H., Gruber A.D., Klopfleisch R. (2018). Validation of digital microscopy compared with light microscopy for the diagnosis of canine cutaneous tumors. Vet. Pathol..

[14] Wei B.R., Halsey C.H., Hoover S.B., Puri M., Yang H.H., Gallas B.D. (2019). Agreement in histological assessment of mitotic activity between microscopy and digital whole slide images informs conversion for clinical diagnosis. Acad. Pathol..

[15] Donovan T.A., Moore F.M., Bertram C.A., Luong R., Bolfa P., Klopfleisch R., Tvedten H., Salas E.N., Whitley D.B., Aubreville M. (2021). Mitotic figures—Normal, atypical, and imposters: A guide to identification. Vet. Pathol..

[16] Meuten D.J., Moore F.M., George J.W. (2016). Mitotic count and the field of view area: Time to standardize. Vet. Pathol..

[17] Bertram C.A., Donovan T.A., Bartel A. (2024). Mitotic activity: A systematic literature review of the assessment methodology and prognostic value in canine tumors. Vet. Pathol..

[18] Bertram C.A., Donovan T.A., Bartel A. (2024). Mitotic activity: A systematic literature review of the assessment methodology and prognostic value in feline tumors. Vet. Pathol..

[19] Evans A.J., Brown R.W., Bui M.M., Chlipala E.A., Lacchetti C., Milner D.A., Pantanowitz L., Parwani A.V., Reid K., Riben M.W. (2022). Validating whole slide imaging systems for diagnostic purposes in pathology guideline update from the College of American Pathologists in collaboration with the American Society for Clinical Pathology and the Association for Pathology Informatics. Arch. Pathol. Lab. Med..

[20] Kiupel M., Webster J.D., Bailey K.L., Best S., DeLay J., Detrisac C.J., Fitzgerald S.D., Gamble D., Ginn P.E., Goldschmidt M.H. (2011). Proposal of a 2-tier histologic grading system for canine cutaneous mast cell tumors to more accurately predict biological behavior. Vet. Pathol..

[21] Dennis M.M., McSporran K.D., Bacon N.J., Schulman F.Y., Foster R.A., Powers B.E. (2011). Prognostic factors for cutaneous and subcutaneous soft tissue sarcomas in dogs. Vet. Pathol..

[22] Trojani M., Contesso G., Coindre J.M., Rouesse J., Bull N.B., De Mascarel A., Goussot J.F., David M., Bonichon F., Lagarde C. (1984). Soft-tissue sarcomas of adults; study of pathological prognostic variables and definition of a histopathological grading system. Int. J. Cancer.

[23] Landis J.R., Koch G.G. (1977). The measurement of observer agreement for categorical data. Biometrics.

[24] Koo T.K., Li M.Y. (2016). A guideline of selecting and reporting intraclass correlation coefficients for reliability research. J. Chropr. Med..

[25] Zuraw A., Aeffner F. (2022). Whole-slide imaging, tissue image analysis, and artificial intelligence in veterinary pathology: An updated introduction and review. Vet. Pathol..

[26] Goldschmidt M.H., Goldschmidt K.H. (2016). Epithelial and melanocytic tumors of the skin. Tumors in Domestic Animals.

[27] Kiupel M. (2016). Mast cell tumors. Tumors in Domestic Animals.

[28] Avallone G., Rasotto R., Chambers J.K., Miller A.D., Behling-Kelly E., Monti P., Berlato D., Valenti P., Roccabianca P. (2021). Review of histological grading systems in veterinary medicine. Vet. Pathol..

[29] Jones N.B., Iwenofu H., Scharschmidt T., Kraybill W. (2012). Prognostic factors and staging for soft tissue sarcomas: An update. Surg. Oncol. Clin. N. Am..

[30] Barboule N., Truchet I., Valette A. (2005). Localization of phosphorylated forms of Bcl-2 in mitosis: Co-localization with Ki-67 and nucleolin in nuclear structures and on mitotic chromosomes. Cell Cycle.

[31] Tabata K., Uraoka N., Benhamida J., Hanna M.G., Sirintrapun S.J., Gallas B.D., Gong Q., Aly R.G., Emoto K., Matsuda K.M. (2019). Validation of mitotic cell quantification via microscopy and multiple whole-slide scanners. Diagn. Pathol..

[32] Bertram C.A., Aubreville M., Marzahl C., Maier A., Klopfleisch R.A. (2019). Large-scale dataset for mitotic figure assessment on whole slide images of canine cutaneous mast cell tumor. Sci. Data.

[33] Bertram C.A., Stathonikos N., Donovan T.A., Bartel A., Fuchs-Baumgartinger A., Lipnik K., van Diest P.J., Bonsembiante F., Klopfleisch R. (2022). Validation of digital microscopy: Review of validation methods and sources of bias. Vet. Pathol..

